# Navigating the Treatment Landscape of Odontogenic Sinusitis: Current Trends and Future Directions

**DOI:** 10.3390/medicina61122175

**Published:** 2025-12-07

**Authors:** Silviu Albu, Alexandra Roman

**Affiliations:** 1 II^nd^ Department of Otorhinolaryngology, Faculty of Dental Medicine, Iuliu Hatieganu University of Medicine and Pharmacy, 400349 Cluj-Napoca, Romania; 2 Department of Periodontology, Faculty of Dental Medicine, Iuliu Hatieganu University of Medicine and Pharmacy, 400012 Cluj-Napoca, Romania; veve_alexandra@yahoo.com

**Keywords:** odontogenic sinusitis, apical periodontitis, endoscopic sinus surgery, dental infection, multidisciplinary care

## Abstract

*Background and Objectives:* Odontogenic sinusitis (ODS) is a particular type of sinus infection induced by dental infections or iatrogenic causes. Although not rare, it is often underrated and sometimes confused with other forms of chronic rhinosinusitis. The aim of this review was to summarize the main diagnostic aspects, microbiological profile, and current options in the therapeutic management of ODS. *Materials and Methods:* Recent studies and consensus statements from both dental and ENT fields were reviewed. The focus was on the ODS diagnostic criteria, the types and the timing of dental and endoscopic treatment approaches, and treatment combinations inducing the best outcomes in ODS. *Results:* ODS usually involves anaerobic bacteria such as *Fusobacterium* and *Peptostreptococcus*. Empirical antibiotics like amoxicillin or amoxicillin–clavulanate are most often used, but antibiotic therapy alone rarely cures the disease. Dental treatment is essential in ODS cases with oroantral fistulas, infected maxillary sinus bone grafts, or implants. However, in these clinical situations, concurrent one-stage dental and endoscopic sinus surgery (ESS) treatment seems to offer the highest success rate, close to 97%. Combined surgery significantly improved ODS treatment outcomes in terms of reduced reintervention rates and recurrence. There is still debate on how wide ESS should be in uncomplicated ODS, but many reports show that maxillary antrostomy alone can be sufficient. In apical periodontitis-related ODS, recurrence after primary ESS is uncommon in the short term. *Conclusions:* ODS management needs cooperation between ENT and dental specialists. Treating the dental underlying infection remains critical to prevent oral or systemic complications. Future research should better define diagnostic criteria, antibiotic use guidelines, and the best timing for combined surgery. New studies on microbiology, immunity, and artificial intelligence could help improve diagnosis and medical care of ODS patients.

## 1. Introduction: Odontogenic Sinusitis—Incidence and Diagnosis

**Figure 1:**
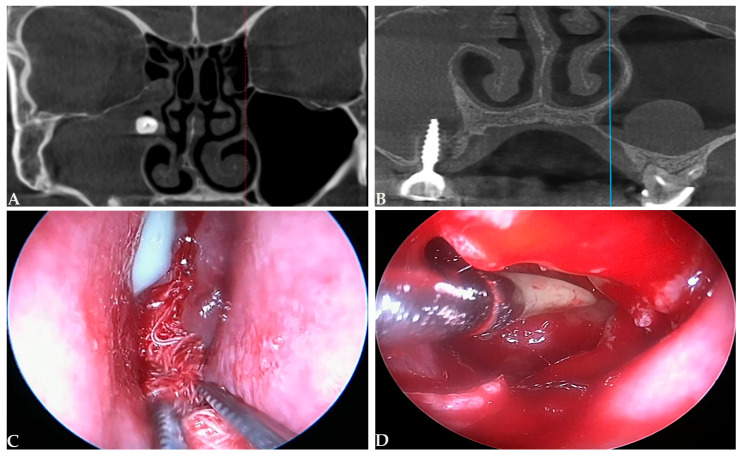
Iatrogenic odontogenic sinusitis induced by dental procedures. CT scans showing maxillary intrasinusal root (**A**) and implant (**B**); Endoscopic sinus surgery showing pus drainage (**C**) and root elimination (**D**). (Photos from the personal archive of the author SA).

Odontogenic sinusitis (ODS) corresponds to a distinctive form of rhinosinusitis that differs profoundly from other types of sinus disease. It describes a maxillary sinus microbial infection starting from neighboring odontogenic infections or iatrogenic injuries accompanying dental procedures [1,2] such as displacement of dental roots or implants into the maxillary sinus during tooth extractions or implant placement (Figure 1). This infection might or might not include the surrounding paranasal sinuses. ODS, a unique type of chronic rhinosinusitis, was previously described, with significant publications dating back to the 1980s and earlier [3,4]. ODS is estimated to account for 25–40% of all cases of CRS involving the maxillary sinus. According to published reports is most often unilateral, accounting for 45–75% of unilateral maxillary sinus clouding detected on computed tomography (CT) scans [1,2,3,4,5]. This disease characteristically develops around the fifth decade of life, impacts both genders equally, and is defined by persistent symptoms lasting approximately six months [6]. The most frequent etiologic factors causing ODS are apical periodontitis and oroantral fistulas [6,7,8].

Lately, an international multidisciplinary consensus statement has been released, promoting a structured diagnostic approach of ODS, highlighting the partnership connecting ENT and dental specialists [1]. As stated by his consensus, the diagnostic process consists of two key steps: the initial suspicion and the subsequent confirmation of ODS [1]: (A) Suspicion: both otolaryngologists and dental clinicians have to detect the essential clinical symptoms that could suggest ODS, providing appropriate referral and prompt assessment; (B) Confirmation: otolaryngologists validate infectious sinus disease by means of nasal endoscopy, while dental specialists recognize the underlying odontogenic problems throughout of focused clinical and radiological evaluations.

Accurate diagnosis therefore depends on the cooperation between both specialties; failure to suspect ODS can easily result in misdiagnosis. Several clinical indicators have been highlighted as particularly suggestive of ODS, such as subjective foul odor, which has been shown as a significant predictor in two prospective cohort studies [8,9]. Compared with chronic rhinosinusitis, ODS patients exhibit a higher rate of purulent discharge on nasal endoscopy [1,2,3,10,11]. Microbiologically, sinus aspirates often reveal elevated proportions of α-hemolytic streptococci and anaerobic bacteria [12,13]. Radiologically, ODS is typically characterized by relative sparing of the posterior ethmoid and sphenoid sinuses on CT, distinguishing it from non-odontogenic rhinosinusitis [1,2,10,11]. All these findings have been more closely related to ODS than with other types of rhinosinusitis [1]. On the CT scan, ODS appears as a unilateral maxillary opacification. However, according to the literature, unilateral disease observed on CT scans is most frequently associated with chronic sinusitis, benign tumors (inverted papilloma, juvenile nasopharyngeal angiofibroma) and malignant tumors (squamous cell carcinoma, lymphoma, sinonasal adenoid cystic carcinoma, sinonasal adenocarcinoma, esthesioneuroblastoma, mucosal melanoma) as well as allergic fungal sinusitis [1,2,3,4,5,6,12]. A recent paper [12] conducted a comparison of findings between chronic sinusitis and malignant tumors in cases with opacified maxillary sinuses. Their research revealed that malignant conditions are typically linked to large masses, extensions beyond the sinus, and masses that cause expansion and thinning of the sinus walls. The presence of these radiographic features should prompt surgeons to consider the possibility of a neoplasm, especially in patients exhibiting unilateral disease. In contrast, benign conditions are generally characterized by mucosal thickening, polyposis, and bony remodeling.

Despite its frequency, ODS has remained underrepresented in the literature, comprising only about 1% of published sinusitis studies during the past two decades [1,2,6,7,8]. In the last ten years, however, research interest has grown substantially, generating a stronger evidence base for both diagnostic criteria and management strategies [6,7,8,9,10]. Particularly within the past three years, several consensus statements have summarized updated recommendations based on systematic reviews and expert panel evaluations [1,2,13,14,15,16,17,18,19].

The purpose of this review is to summarize current insights regarding ODS diagnosis and management, emphasizing the timing and efficacy of antibiotic therapy and dental interventions, whose outcomes directly influence decisions about when to perform endoscopic sinus surgery (ESS) [14,15,16,17,18,19].

## 2. Methods

Publications from January 2008 to August 2025 were considered for this narrative review. Searches were conducted in PubMed, Web of Science and the Cochrane Library using a combination of MeSH terms and related keywords linked by Boolean operators (AND, OR) to combine concepts and synonyms. The main terms included: “*Odontogenic sinusitis*”, “*Endoscopic Sinus Surgery*”, “*Root canal therapy*”, “*Endodontic treatment*”, “*Apical periodontitis*”, “*Dental Implant*”, “*Oroantral fistula*”, “*Periimplantitis*”. Quotation marks were used for exact phrases, and the asterisk (*) served as a truncation symbol to capture word variations.

The inclusion criteria encompassed case reports, case series, retrospective reviews, or randomized controlled trials. Included in the analysis were adult patients with odontogenic sinusitis (over 18 years old) and English language articles. The exclusion criteria included textbook chapters, review articles, non-English publications, articles lacking clinical data. Any conflicts were resolved through discussion. Titles and abstracts obtained from the database search were screened independently by both authors (S.A., and A.R.), and full texts were downloaded in case of agreement. Additionally, the reference lists of included studies were examined to identify further relevant articles.

Data from the included articles were extracted and entered into a Microsoft Excel database. The documentation included the publication title, first author, year of publication, country, study type, surgical technique used, dental pathology, imaging and endoscopic findings, outcomes, and complications. Data analysis was mainly descriptive due to the nature of the collected information.

A total of 40 articles were initially identified, comprising 10 systematic reviews and 30 clinical studies. Of the systematic reviews, two were excluded for not meeting the inclusion criteria and an additional two were duplicates. Among the clinical studies, only 24 met the eligibility criteria. Consequently, the final analysis included six systematic reviews and 24 clinical studies.

## 3. Dental Pathology Triggering Odontogenic Sinusitis

A systematic review published in 2014 and including 674 ODS patients identified iatrogenic sinusitis in 65.3% of cases, apical periodontal pathology in 25.1%, and periodontitis in 8.3% [6]. As previously mentioned, most authors regard iatrogenic factors as the leading cause of ODS [1,6]. Such mechanisms may result from sinus floor perforation during dental extractions or endodontic procedures, dental ankylosis, misplaced dental implants, orthognathic or pre-prosthetic maxillary surgery, as well as sinus lift and sinus graft interventions [1,2,3,4,7,11,12,13,14,15]. In all these settings, infection arises through bacterial colonization from the oral microbiota [12,13,14].

Before considering medical, dental, or surgical (ESS) treatment modalities for ODS, it is essential to recognize that various dental aetiologies may contribute to the disease, and not all require direct dental intervention [15,16]. Craig et al. [16,19] proposed classifying dental causes into treatable and non-treatable categories.

Treatable dental sources include: apical periodontitis, with or without periapical lesions (endodontic infection), apical periodontitis following failed root canal therapy, periodontitis (periodontal disease), oroantral fistulas (OAF), infected dentigerous cysts, infected maxillary sinus bone grafts or dental implants, and foreign bodies retained in the alveolar ridge after dental procedures [16,17,18,19].

Conversely, non-treatable dental conditions are those where the initial dental source is no longer active—for instance, transient oroantral communications (OACs) after dental procedures, persistent ODS despite a successful root canal therapy, non-infected dental implants or sinus bone grafts, and foreign bodies within the sinus that are not associated with an active infection or OAF, which can thus be managed exclusively by ESS [16,19].

## 4. Management Options in Odontogenic Sinusitis

### 4.1. Antibiotic Therapy in Odontogenic Sinusitis

In patients with ODS the nasal microbiome is mainly composed of anaerobic bacteria, most often Gram-negative bacilli, such as *Fusobacterium*, *Prevotella*, *Peptostreptococcus* and *Porphyromonas* [18,19,20]. Numerous studies have shown that these bacterial genera are more prevalent in ODS patients compared to those with chronic rhinosinusitis (CRS) [13,14,15,16]. The odontogenic focus enables pathogens to invade the sinus, which is typically a sterile cavity, often serving as a reservoir of bacteria that perpetuates the infection. Dental diseases and treatments can introduce oral pathogens into the maxillary sinus [13,14,15,16]. Given that these oral pathogens are predominantly anaerobic (with an aerobe to anaerobe ratio in the oral flora ranging from 1:10 to 1:100), it is not surprising that anaerobic bacteria play a crucial role in ODS [14]. The presence of anaerobic bacteria in the nasal microbiome of individuals with ODS may indicate tissue hypoxia or suggest that the unique microenvironment within the mucus allows for limited oxygen availability, creating a favorable niche for the survival of anaerobic bacteria [16]. Anaerobic bacteria are sometimes resistant to common antibiotics used for treating sinus infections, making it essential to consider their presence when developing treatment plans. It has been demonstrated that the presence of β-lactamase-positive anaerobic bacteria is consistently higher in ODS as opposed to other forms of sinusitis [13,14,15,16].

Conversely, several reports describe in ODS the accompanying occurrence of aerobic microorganisms such as *alpha-haemolytic Streptococcus*, *microhaemolytic Streptococcus*, and *Staphylococcus aureus* [21]. Up to now more than 158 bacterial species have been identified in relation to ODS, along with numerous fungal strains. Inside infested root canals, bacteria susceptible produce secondary periapical lesions include *Propionibacterium*, *Peptostreptococcus*, *Fusobacterium*, *Dialister*, *Prevotella*, other oral anaerobes, and *Candida* species—most notably *Candida albicans* [20,21,22].

The literature addressing endodontic infections suggest that these bacterial species display significant susceptibility to penicillin, amoxicillin, and amoxicillin–clavulanate (90–100%), variable sensitivity to clindamycin (73–96%), and reduced responsiveness to metronidazole (45–55%), tetracyclines (60%), and erythromycin (55%) [19,23,24,25,26,27,28]. In vitro investigations have also indicated that fluoroquinolones are effective against a wide array of oral pathogens [19,27]. Nonetheless, there are no published data directly examining antibiotic resistance patterns in proven ODS. According to the bacteriological results offered by related research, the most reasonable approach for empirical therapy is to start the treatment with amoxicillin or amoxicillin–clavulanate as first-line options, and to take into account moxifloxacin or levofloxacin in patients allergic to penicillin [19,24,25,26,27,28].

As described in various case series and cohort studies, patients repeatedly go through multiple unsuccessful courses of oral antibiotics before obtaining definitive dental treatment or ESS. This recurrent failure highlights that antibiotic therapy alone seldom provides adequate resolution of ODS [2,16,18,19]. In endodontic disease specifically, reliance solely on antibiotics without appropriate dental management is regarded as ineffective [28]. An expert multidisciplinary panel has accordingly concluded that the use of oral antibiotics as monotherapy in cases of ODS secondary to treatable dental pathology is not an appropriate or curative approach [1,16,19].

Theoretically, the success of antimicrobial therapy could be higher when there is no active infectious dental focus [19,20,21,22,23,24,25,26,27,28,29]. Nonetheless, most published studies include patients with both treatable and non-treatable odontogenic sources, and antibiotics alone have failed to resolve sinus disease even in the absence of ongoing dental infection [19,20,21,22,23,24,25,26,27,28]. Additional research is required to determine whether a subset of ODS cases might respond favorably to antimicrobial therapy alone—particularly in those lacking a correctable dental cause.

Although oral antibiotics rarely eradicate ODS completely, they still have supportive roles. In uncomplicated presentations, with or without a treatable dental focus, antibiotics may alleviate sinus or dental symptoms until definitive treatment is completed. In more complex or intricate cases, antibiotic treatment can aid in securing orbital or intracranial complications, especially when used alongside surgical drainage as necessary [18,30,31]. Additionally, prophylactic or postoperative antibiotics are often recommended straight away following dental or sinus surgery, while substantial evidence for this practice is still lacking [19].

Future clinical studies should aim to compare outcomes between patients who receive postoperative antibiotics and those who do not, both after dental interventions and ESS [19]. Likewise, optimal treatment duration—before or after surgical management—has yet to be defined through controlled research in the context of ODS.

### 4.2. Dental Treatment in Odontogenic Sinusitis

For ODS associated with treatable dental pathology, eliminating the underlying dental infection is essential for achieving complete resolution. Dental therapy is appropriate in cases where ODS originates from pulpal, periapical, or periodontal diseases, and may involve root canal therapy, apicoectomy, or tooth extraction as indicated.

In 2018, the American Academy of Endodontics released a position statement on maxillary sinusitis of endodontic origin, recommending that dental treatment should constitute the first line of management, with ESS reserved for refractory cases [32]. The document cited multiple case reports and series describing complete resolution of maxillary ODS following appropriate endodontic therapy [32]. Supporting this view, Tomomatsu et al. [33] evaluated 39 patients with maxillary ODS who underwent primary endodontic treatment or extraction, combined with three months of oral antibiotics. Patients were monitored for sinusitis resolution, defined by either the absence of inflammation on CT or the disappearance of clinical symptoms. Reported symptoms included pain, swelling, headache, and a feeling of sinus pressure, though the study did not specify which symptoms resolved or their duration. Other typical sinusitis manifestations were also not documented. Based on the authors’ criteria, 20 patients (51.2%) achieved full resolution following dental treatment alone, while 19 remained symptomatic and required ESS, which successfully resolved their disease. The time to resolution was not reported, and follow-up for the dental treatment group was limited to three months post-antibiotic therapy, with no long-term outcomes available [19].

Longhini and Ferguson [34] also presented a case series of 21 ODS patients, 19 of whom experienced resolution after dental extractions. Their findings supported the concept that dental management should precede ESS, although they did not include information about the timeframe of recovery, nor did they assess clinical improvement, quality of life, or endoscopic outcomes following intervention [19].

Further indirect evidence reinforcing the need to address dental pathology before ESS comes from studies demonstrating that undiagnosed odontogenic infections may lead to surgical failure. Wang et al. [35] conducted a retrospective chart review of 55 patients with ODS, assessing treatment outcomes for 31 of them. The therapeutic approaches included antibiotics alone, dental treatment, ESS, or a combination thereof. Among these patients, 21 achieved resolution: seven (33%) after ESS alone, seven (33%) with concurrent ESS and dental therapy, two (10%) with dental therapy alone, two (10%) with antibiotics alone, two (10%) with ESS following failed dental treatment, and one (5%) with antibiotics after unsuccessful dental management. Resolution was defined as the absence of inflammation on nasal endoscopy or sinus CT, with an average follow-up of five months. Symptoms of sinusitis were not discussed. The authors concluded that while most patients improved, the heterogeneity of treatment combinations prevented firm conclusions regarding the optimal timing of interventions.

Two recent prospective studies [36,37] have reported improved success rates for dental extractions in resolving ODS associated with apical periodontitis compared to earlier research. Yoo et al. [36] prospectively followed 33 patients with ODS due to dental caries and periapical abscesses. Of these, 22 (67%) achieved complete resolution through dental and medical management, whereas 11 (33%) required ESS after unsuccessful conservative therapy. Multivariate analysis indicated that smoking and higher Lund–Mackay CT scores were predictors of treatment failure, necessitating subsequent ESS. The authors concluded that two-thirds of ODS cases secondary to caries and periapical abscesses can be effectively managed with dental and medical treatment alone, recommending this approach as the initial step. In contrast, for smokers and patients with severe sinus changes on CT, they advocated earlier ESS [36].

In another large prospective study, Simuntis et al. [37] examined 96 patients with ODS secondary to apical periodontitis, reporting a 77% success rate following dental extraction alone; the remainders required subsequent ESS. Persistent foul odor two weeks post-extraction was identified as a clinical indicator of treatment failure.

Nonetheless, these investigations have several limitations. They primarily included patients with isolated maxillary sinus involvement, characterized by variable mucosal thickening, but lacked pretreatment nasal endoscopy, meaning some participants may not have had true infectious sinusitis. Additionally, the severity of disease among included patients appeared lower than that described in broader literature, where approximately 70% of ODS cases involve the ethmoid sinus and 40% the frontal sinus [1,16,19,38]. The extent of sinus involvement, reflected by higher Lund–Mackay scores, correlates inversely with the success of primary dental management [36]. Thus, the relatively high resolution rates reported by Simuntis et al. [37] may partially result from a less extensive disease burden in their study population.

However, the evidence cited in that position statement primarily originated from small case reports and limited case series, many of which described patients with mild mucositis rather than purulent odontogenic sinusitis. More recently, a study failed to confirm the efficacy of dental therapy alone in resolving ODS [39]. This investigation included 68 symptomatic patients with periapical lesions. Each patient was evaluated by both an otolaryngologist and a dentist through clinical interviews, nasal endoscopy, cold pulp testing, and tomographic imaging. Participants were followed prospectively for at least 12 months, receiving nasal corticosteroids, saline irrigations, and root canal therapy.

The outcomes showed that only 9 patients (13%) improved with conservative management, whereas 59 patients (87%) required surgical intervention. Improvement following medical therapy and root canal therapy correlated with younger age (*p* = 0.043) and a greater distance between the apex of the periapical lesion and the floor of the maxillary sinus (*p* = 0.003). The study concluded that a larger number of affected roots and proximity of the periapical lesion apex to the sinus floor were significant predictors of medical treatment failure. Once bone destruction extended into the maxillary sinus, combined tooth extraction and ESS were required for complete disease resolution [39].

### 4.3. Endoscopic Sinus Surgery in Odontogenic Sinusitis

ESS is considered mandatory in the management of complicated ODS. The term “complicated odontogenic sinusitis” refers to infections that extend beyond the maxillary sinus, affecting structures such as the orbit, facial soft tissues, intracranial compartments, bone, or other systemic regions [18,30,31].

According to international guidelines for managing complicated non-odontogenic rhinosinusitis, patients should receive intravenous antibiotics for 48 h before proceeding with ESS—unless there is visual loss or an extrasinus abscess amenable to transnasal drainage [18]. Abscesses inaccessible through the nasal cavity should be managed via appropriate external drainage. If the patient’s condition deteriorates or fails to improve within this 48 h interval, ESS becomes indicated [18].

A recent systematic review evaluated the epidemiological, clinical, and therapeutic features of complicated ODS [30]. Approximately 70% of such cases involved orbital complications. Remarkably, only 23% of publications addressing complicated ODS appeared in otolaryngology journals. Among cases with orbital or intracranial involvement, around 80% occurred in adults, 75% of whom were male. The most common underlying dental cause was apical periodontitis of maxillary molars. No significant relationship was identified between the extent of sinusitis and the likelihood of orbital or intracranial spread. Microbiologically, infections were characterized by high loads of anaerobic and α-hemolytic streptococcal species. Treatment generally combined systemic antibiotics active against both aerobic and anaerobic organisms with surgical procedures addressing the complications (orbital or intracranial) and eliminating the odontogenic or sinonasal source [30].

It is important to note that the urgency and timing of ESS in complicated ODS, compared with complicated rhinosinusitis, have not been thoroughly studied; therefore, surgical judgment remains essential [20]. A systematic review reported that 82% of patients (32 of 39) with treatable dental pathology underwent dental extractions during hospitalization, although most of these studies were authored by oral surgeons [30]. In contrast, a multicenter retrospective analysis published by rhinologists found that among 45 patients with complicated ODS treated with ESS, none underwent dental procedures during admission, yet no recurrences were observed during follow-up [31]. Ideally, the dental focus should be managed during hospitalization, though this is not always feasible.

In iatrogenic ODS, surgical management is frequently required [40]. For instance, Kim et al. [41] investigated 19 cases of ODS caused by dental implants, finding that only 21% responded to conservative therapy, while 79% required ESS. Similarly, Chen et al. [42] reported that 15 of 18 patients with implant-related ODS needed surgical intervention. In a 2022 retrospective study, Shin et al. [43] observed a rising incidence of iatrogenic ODS, with two-thirds of 33 patients requiring ESS, a notably higher rate than previously documented. These data highlight the growing role of ESS as a key therapeutic option, particularly in iatrogenic ODS. Moreover, higher Lund–Mackay scores were strongly correlated with an increased need for surgical treatment.

### 4.4. The Treatment Sequence of ESS and Dental Therapy in Odontogenic Sinusitis

**Figure 2:**
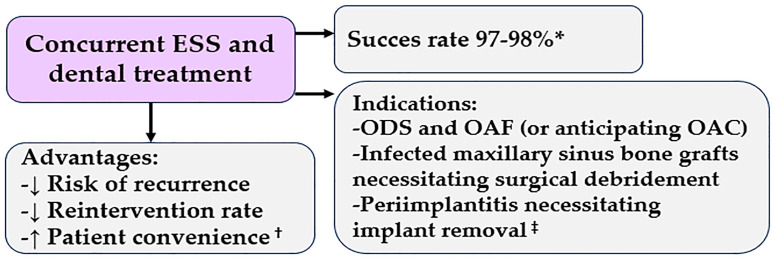
The Treatment sequence of endoscopic sinus surgery and dental therapy in odontogenic sinusitis. ESS, endoscopic sinus surgery; ODS, odontogenic sinusitis; OAC, oroantral communication; OAF, oroantral fistula, * [47,48], ^†^ [49], ^‡^ [50,51,52].

Because dental therapy alone fails to achieve resolution in a considerable proportion of ODS cases, primary ESS may represent a valid therapeutic alternative (Figure 2). Approximately 85–90% of ODS patients present primarily with sinus-related symptoms, whereas only up to 40% report dental pain [44,45]. One investigation even showed that around 85% of patients with ODS initially consult a rhinologist for sinus complaints rather than seeking dental evaluation [46].

Although ODS patients typically present first to otolaryngologists, symptom intensity can vary widely. When individuals exhibit minimal or absent sinus symptoms, referral to dental specialists for definitive dental management remains the preferred course [16,19]. In contrast, when sinus manifestations predominate, current evidence supports the use of primary ESS as a reasonable initial step [19].

Craig et al. [47] prospectively studied 37 symptomatic ODS patients unresponsive to medical therapy. Participants could choose between primary dental intervention or ESS. Eleven patients underwent dental therapy first, whereas 26 opted for ESS. At each visit, both before and after intervention, the following parameters were recorded: Sino-Nasal Outcome Test-22 (SNOT-22) scores, presence or absence of cardinal sinusitis symptoms, and endoscopic findings such as edema, polyps, and purulence. Kaplan–Meier survival analysis was used to determine the time to symptom resolution. Results demonstrated that primary ESS achieved faster improvement in sinus symptoms, SNOT-22 scores, and endoscopic outcomes than primary dental therapy. The authors concluded that ESS should be considered the first-line treatment for symptomatic ODS, with dental management performed as required thereafter [47].

In a complementary study, Choi et al. [53] observed no significant difference in outcomes between patients who underwent primary versus secondary ESS, provided that both the dental and sinonasal sources were ultimately treated. Similarly, a multidisciplinary consensus has affirmed that primary ESS is an appropriate approach for ODS cases accompanied by marked sinusitis symptoms.

Nonetheless, primary dental therapy remains a valid initial choice in symptomatic patients with treatable dental pathology, provided that treatment planning involves a shared decision among the patient, otolaryngologist, and dental specialist. This discussion should balance the risk of dental treatment failure or delayed sinus resolution against the potential surgical risks of ESS [19,44,45,46,47,53].

An important question concerns the value of concurrent ESS and dental treatment, as several large case series have reported overall success rates of 97–98% [48,50,51,54]. However, there are no direct comparative studies between concurrent and sequential treatment strategies across different odontogenic etiologies, making it difficult to establish specific indications for simultaneous management.

Retrospective analyses from Felisati and colleagues [52] at the University of Milan suggest that concurrent ESS and dental treatment may be particularly beneficial in conditions such as: ODS associated with OAFs or anticipated OACs during tooth or implant removal; infected maxillary sinus bone grafts requiring surgical debridement; and peri-implantitis necessitating implant removal.

These recommendations arose from observations that failure to address dental pathology during ESS often resulted in delayed healing or recurrence of ODS. The concept proposed by Felisati [52] was subsequently validated in a prospective study by Saibene et al. [49].

Expanding the patient sample to 364 individuals, Kocum et al. [48] further supported this combined approach, reporting a 97% success rate and consistent intraoral and intranasal healing within three months post-surgery. Based on these findings, the authors anticipated complete resolution of ODS in all patients within the same time frame.

Additional evidence comes from Gata et al. [55], who examined 31 ODS patients with OAFs. They found that those treated with concurrent ESS and OAF closure experienced resolution in half the time compared to patients undergoing OAF closure alone (10 vs. 20 days, *p* = 0.001). Consequently, for ODS cases involving OAFs, concurrent ESS and fistula closure are considered optimal. The benefit likely stems from the reduction in sinus pressure and decrease in bacterial load within the maxillary sinus.

Regardless of symptom severity, it is recommended that ODS patients with OAFs, infected bone grafts, or contaminated dental implants requiring removal undergo ESS, either as a primary intervention followed by oral surgery or simultaneously, when feasible [55].

A recent study has sought to directly compare single-stage combined surgical management—performed collaboratively by otolaryngology and maxillofacial surgery teams—with non-combined approaches [56]. This retrospective observational analysis, which included 96 patients treated surgically for ODS between January 2019 and December 2024, classified participants according to both etiology and surgical strategy (combined vs. non-combined). The results demonstrated that combined surgery significantly reduced reoperation rates (*p* = 0.003), particularly in cases associated with periodontal or endodontic infections (*p* = 0.002). These findings suggest that single-stage combined intervention effectively lowers the risk of recurrence and the need for additional surgery in ODS.

Furthermore, in staged treatment scenarios, initiating therapy with dental or maxillofacial intervention before otolaryngologic surgery was associated with better postoperative outcomes [56].

One practical advantage of performing primary or concurrent ESS alongside oral surgical management is the prompt elimination of purulent sinus infection, which can otherwise interfere with OAF closure. Further studies are needed to determine whether oral surgical procedures alone, without ESS, can achieve comparable outcomes in such cases. Another notable benefit of concurrent ESS with dental intervention is improved patient convenience—a single combined procedure reduces the need for multiple hospital visits. Future research will be instrumental in identifying which subsets of ODS patients benefit most from simultaneous ESS and dental treatment.

An additional aspect worth exploring concerns the recurrence timeline of sinusitis following primary ESS in ODS related to treatable dental pathology. Two studies have indicated that purulent sinusitis does not commonly recur soon after ESS in cases caused by apical periodontitis of endodontic origin. Zhao et al. [57] evaluated 48 patients who underwent ESS for ODS, of whom 35 had untreated apical periodontitis. During a six-month follow-up, only one patient (2.9%) with apical periodontitis experienced recurrence of purulent sinusitis. Similarly, Craig et al. [47] observed that among 25 ODS patients with apical periodontitis who underwent primary ESS, 14 proceeded with dental treatment while 7 declined further dental care. None of the latter developed recurrent sinusitis over an average follow-up of seven months.

Although these studies were limited by small sample sizes and short observation periods, their findings suggest that ODS secondary to apical periodontitis may not frequently relapse in the short term after ESS. Consequently, for patients reluctant to undergo immediate dental treatment for apical periodontitis, primary ESS followed by endoscopic monitoring may represent a reasonable approach. The results reported by Zhao et al. [57] and Craig et al. [47] also suggest that certain dental infectious lesions might not inherently trigger sinusitis recurrence. The rationale is that the infection does not re-enter the maxillary sinus, or the dental problem resolves on its own or following minimal treatment. Nevertheless, this outcome likely applies only to apical periodontitis-related ODS, and should not be generalized to cases involving OAFs, infected bone grafts, or dental implants, where the risk of persistent infection remains high.

In conclusion, even though some forms of ODS can resolve following ESS alone, it remains strongly advisable to pursue definitive dental management whenever a treatable odontogenic focus exists. Primary dental treatment may fully eradicate ODS or, at minimum, reduce the likelihood of recurrence when ESS is performed first. Furthermore, addressing the underlying dental infection—irrespective of sinus symptom resolution—is essential to prevent potential local or systemic dissemination of infection beyond the sinonasal region.

### 4.5. Extent of Endoscopic Sinus Surgery in Odontogenic Sinusitis

A substantial proportion of patients with ODS will eventually require ESS as part of their multidisciplinary management. Determining the optimal surgical extent for these patients remains an important yet insufficiently explored issue. A recent multidisciplinary consensus statement addressed the surgical approach to ODS, differentiating between uncomplicated and complicated cases [16].

In complicated ODS, where there is risk of visual loss or neurological complications, consensus dictates that all involved sinuses should be opened during ESS [16,17,18,19]. However, no clear agreement was reached regarding the extent of surgery required in uncomplicated ODS. The absence of consensus is partly explained by findings from two prospective studies reporting complete (100%) resolution of ODS three months after maxillary antrostomy (MA) alone, even in patients with frontal sinus involvement [58,59].

In one of these investigations, Ungar et al. [58] conducted a prospective study including 25 ODS patients, demonstrating full resolution after MA alone for cases involving the maxillary, anterior ethmoid, and frontal sinuses. Disease improvement was confirmed by nasal endoscopy at three months postoperatively and by SNOT-22 evaluations at three to six months. The same team later published an expanded series of 45 ODS patients, again reporting 100% resolution following MA alone in cases with frontal sinus involvement, as assessed by both endoscopic and symptomatic criteria [59].

This view, however, has been challenged by Kocum et al. [48], whose 18-year retrospective study included 364 adult patients. Their findings suggested that in the presence of purulence within the ethmoid, frontal sinus, or nasofrontal recess, and particularly in immunocompromised patients at risk of complications, it is advisable to perform ethmoidectomy and noninvasive frontal sinusotomy. Adequate opening of affected sinuses to restore drainage and ventilation may not only prevent complications but also accelerate recovery.

To address these differing perspectives, Craig et al. [60] designed a multicenter prospective study evaluating outcomes of MA alone versus complete ESS. The study enrolled 70 patients with uncomplicated ODS involving the maxillary, anterior ethmoid, and frontal sinuses, as confirmed by CT imaging. Thirty-five patients underwent MA alone, while the remaining 35 received complete ESS. One key finding was that although anterior ethmoid purulence resolved more slowly after maxillary antrostomy alone, both groups experienced similar symptomatic improvement, particularly regarding foul odor reduction and SNOT-22 score decreases, at all postoperative intervals.

A plausible explanation for this comparable symptomatic improvement, despite delayed anterior ethmoid clearance, lies in the fact that the majority of preoperative purulence accumulates in the maxillary sinus, the largest of the paranasal sinuses. Therefore, drainage achieved through MA alone removes most of the infectious material, yielding substantial relief. In the MA group, anterior ethmoid purulence resolved completely by the two-month postoperative follow-up.

From another standpoint, some surgeons advocate for complete ESS in these situations, emphasizing faster purulence clearance. Nonetheless, several practical considerations must be acknowledged when opting for an extensive procedure, particularly concerning frontal sinus surgery. Endoscopic approaches to the frontal sinus remain technically challenging regardless of inflammatory severity [61]. Moreover, the operative field in ODS is often obscured by copious purulence and highly vascularized mucosa, increasing the risk of significant intraoperative bleeding. Indeed, estimated blood loss has been shown to be greater in complete ESS compared with MA alone. When key anatomical landmarks are masked, the likelihood of incomplete dissection or iatrogenic complications rises, potentially resulting in postoperative stenosis of the frontal recess [19,60,61].

As emphasized by Craig et al. [19], there are currently no data on whether isolated MA can achieve resolution in ODS cases involving the posterior ethmoid or sphenoid sinuses. In such instances, it is therefore prudent to fully open at least the maxillary, anterior and posterior ethmoid, and sphenoid sinuses. Furthermore, in regions where balloon sinuplasty is available, outcomes for ODS following this technique have yet to be systematically evaluated, underscoring a need for further investigation.

Finally, although unilateral ODS rarely spreads contralaterally, in cases where infection extends across the midline, it is reasonable to ensure adequate surgical drainage of affected contralateral sinuses to prevent persistence or recurrence.

### 4.6. Recurrence Rates in Odontogenic Sinusitis

Currently, there is a lack of systematic evaluation regarding the prognosis of ODS treatment. The literature shows variability in the incidence of recurrence, influenced by the type of study and patient cohort. Galli et al. [50] reported a recurrence rate of 5.9% in a cohort of 34 patients, all of whom received combined functional ESS and oral surgery. Zirk et al. [28] documented two surgical revisions in a group of 121 patients (1.6%). Molteni et al. [51] found a recurrence rate of less than 1% after evaluating 480 patients. Galli et al. [50] identified diabetes mellitus and nicotine as potential risk factors for recurrence, suggesting that tobacco use triggers the release of catecholamines, leading to peripheral vasoconstriction, tissue ischemia, and delayed healing. Similarly, patients with diabetes mellitus are more prone to postoperative complications due to increased susceptibility to chronic inflammation.

In a recent study, Sakkas et al. [62] aimed to identify factors influencing recurrence after surgical treatment of ODS in an oral and maxillofacial surgery clinic over a 7-year period. A multivariable analysis was conducted to explore associations between patient age, causative focus, surgical access for sinus revision, multilayer closure with a buccal fat pad, inferior meatal antrostomy for temporary sinus drainage, and recurrence of sinusitis. A total of 164 patients with a mean age of 51.7 years were included, and recurrence of sinusitis was observed in nine patients (5.48%) within 6 months post-surgery. No significant correlations were found between these factors and recurrence development (*p* > 0.05). However, patients with a history of antiresorptive-related osteonecrosis of the jaw showed a significant tendency toward recurrence (*p* = 0.0375).

The relationship between timing of ESS in cases of ODS has been recently addressed in the literature. Yoo et al. [36] reported that 67% of 33 ODS patients recovered after receiving both medical and dental treatments, with only 33% needing additional ESS. In contrast, some authors advocated prioritizing ESS. Abdulkader et al. [63] conducted a retrospective cohort study that demonstrated that ESS followed by dental treatment led to a shorter overall treatment duration compared to the reverse order of treatment. In the study conducted by Wang et al. [35], among the 21 patients who recovered, 33% (7) resolved with ESS alone, 33% (7) resolved with a combination of ESS and dental treatment, and 10% (2) resolved with dental treatment alone. However, it was also noted that 29% of ODS patients were refractory to ESS alone.

In a recent study, Zhao et al. [57] explored the need for subsequent dental treatment in patients with ODS following ESS. The study included 48 patients, of which 35 cases (72.9%) were attributed to dental diseases, specifically periapical periodontitis, while 13 cases (27.1%) resulted from iatrogenic factors, such as tooth extraction or implantation. The overall treatment success rate was 97.9%, with success rates for the dental disease group at 97.1% and the iatrogenic group at 100%, showing no significant difference (X^2^ = 0.329, *p* = 0.729). Notably, none of the patients in the dental disease group received treatment for the causative teeth within 6 months post-surgery, except for one patient who underwent tooth extraction 6 months after the procedure.

The significant variability in the results of these studies may be attributed to differences in patient populations, varying inclusion criteria for ODS, and discrepancies in the severity of dental diseases and sinusitis. For instance, ODS patients who recovered solely from dental treatment might have a lower burden of sinus disease, while those with mild dental issues may fully recover with ESS alone.

## 5. Future Directions in the Management of Odontogenic Sinusitis

Recent literature has underlined the relevance of correctly including ODS into existing sinusitis management guidelines, supported by clinical studies that recognize its unique pathophysiology and treatment factors [60]. Efficient management depends on close collaboration between otolaryngologists, dental practitioners, and radiologists, demanding the establishment of interdisciplinary clinical networks for complete diagnosis and organized care [1,2,19,64].

However, the true prevalence of ODS remains uncertain, primarily because of the lack of widely recognized diagnostic standards and the absence of specific ICD codes for this disease [65]. A more precise understanding will require well-designed prospective studies highlighting clearly defined inclusion and exclusion criteria, distinguishing ODS from pure maxillary sinus mucositis [64,65].

From an educational perspective, several authors have argued for the urgent inclusion of evidence-based ODS data within national and international otolaryngology and dental curricula. Present educational programs inadequately represent ODS, resulting in low awareness among clinicians. Updating medical and dental school curricula, residency programs, and continuing professional education courses is crucial to guarantee that ODS is properly diagnosed and managed in the clinical practice [64,65].

Clinically, creating organized communication pathways and uniform referral processes between dental and ENT practitioners is decisive to enhance patient outcomes and decrease treatment delays [1,16,19]. Otolaryngologists should refer patients to the appropriate dental specialist corresponding to the underlying dental pathology, whereas dental professionals must work in partnership with ENTs qualified in managing ODS [1,2,3,4,5,6,7,8,9,10,11,12,13,14,15].

Future research priorities include determining the accurate ODS epidemiology and its correlation with diverse dental pathologies [19,64].

More comprehensive studies are also needed to identify the most effective dental interventions for ODS arising from apical periodontitis (endodontic disease) or OAFs [16,19,64]. Dental specialists should quantify the success rates of root canal therapy and extractions in resolving ODS, particularly in cases involving periapical abscesses. Importantly, these investigations should exclude patients presenting only with maxillary sinus mucosal thickening rather than true ODS [64,65,66].

There is also a notable knowledge gap regarding ODS management in OAFs. While some clinicians advocate for concurrent ESS and OAF closure [67], others suggest that small fistulas may close spontaneously after ESS alone. This phenomenon, however, remains unverified, presenting an opportunity for further research into the frequency and mechanisms of spontaneous OAF closure following ESS [64].

In periodontics, future studies should clarify the stage and grade of periodontitis that predispose to ODS, distinguishing them from cases of simple sinus mucosal thickening, and evaluate whether periodontal therapy can effectively resolve ODS in selected patients [65]. A similar need for clarity applies to implantology, particularly regarding the severity of peri-implantitis capable of inducing ODS. Determining when sinus bone grafts and implants should be removed to ensure resolution of infection will be an important step toward evidence-based management [64].

Additional analyses should also report the optimal timing, duration, and efficacy of oral antibiotic treatment provided before and after dental or sinus surgeries. Accomplishing this assignment will necessitate multicenter studies implying both dental and ENT teams. Dental professionals should assess whether antibiotics administered following root canal treatments or extractions enhance sinusitis resolution rates. The previous statement disagrees with traditional dental guidelines that advise against the use of antibiotics solely for these procedures.

Recognizing that ODS presents a different clinical context, dentists must coordinate with otolaryngologists to avoid delays or omissions in antibiotic therapy—factors that may explain the low success rates of dental-only treatment reported in the literature [60].

Another clinical question involves patients who continue to experience purulent sinusitis after extractions. Research is needed to identify the most effective antibiotic regimens and durations for such cases, or alternatively, to determine whether early ESS provides faster and more reliable relief [61].

Pediatric ODS also remains underexplored. Further work is required to elucidate differences in pathophysiology and therapeutic response between children and adults [68]. On a molecular level, basic science studies focused on inflammatory pathways and microbial profiles could clarify why only a subset of patients with dental disease develop ODS. A recent investigation of the inflammatory endotype of ODS found elevated levels of IFNγ, TNFα, IL-6, IL-8, IL-10, IL-27, and CXCL9, but comparable IL-17 levels between ODS and control groups [69]. These findings suggest that ODS involves increased innate and Th1-mediated immune activity. Further immunopathological studies should perfect ODS pathogenic networks and guide future therapeutic strategies.

Lastly, the integration of artificial intelligence (AI) into ODS management represents an emerging frontier. A recent study evaluated the performance of large language model AI systems, specifically the Chat Generative Pre-Trained Transformer (ChatGPT) 3.0, in addressing complex otolaryngological scenarios including ODS [70]. While newer versions of large language models showed promise in enhancing evidence-based decision-making, marked discrepancies persisted between AI-generated outputs and expert clinical reasoning. Thus, current of large language models cannot yet substitute specialist judgment. Future research should focus on refining AI capabilities to support multidisciplinary diagnostic and management processes in ODS as technology continues to evolve.

## 6. Limitations

A potential limitation of our study concerns the search terms described in the Section 2. The omission of certain search terms could lead to the failure to identify all possible combinations of terms related to the subject addressed.

This review paper as a narrative review has its distinct limitations. In this study, there is a potential for selection bias in the articles included and thus subjectivity in the conclusions drawn. To mitigate this risk, two independent reviewers were involved at every stage. Most of the studies included in the analysis display differences in patient populations, numerous inclusion criteria for ODS, and discrepancies in the severity of dental diseases and sinusitis. Because of this mentioned heterogeneity, an adequate and robust analysis is not feasible. Since the percentage of ODS recurrences following ESS is reduced, the retrospective nature of the studies and the small number of cases, a powerful prediction of ESS failures is unlikely. Furthermore, our investigation lacks a formal evaluation of the quality of the included studies, which may impact the reliability of the findings. Unfortunately, there are currently no tools available to quantify the risk of bias in case reports and case series. Nonetheless, a modified version of the Newcastle-Ottawa scale (NOS) has been used as an instrument for risk of bias assessment by other investigators [71,72]. Our results may be specific to the context and not generalizable because of the selective nature of the studies included.

This limitation is typical for this type of review, and we discuss it in the thematic analysis of the identified studies.

## 7. Conclusions

In ODS cases other than those linked to apical periodontitis—such as oroantral fistulas, infected bone grafts, or contaminated dental implants—primary dental management remains indispensable.

For ODS secondary to apical periodontitis, short-term recurrence appears uncommon following ESS performed as the primary intervention.

ESS, accompanied by subsequent endoscopic follow-up, can serve as a practical interim solution in cases where immediate dental treatment is deferred.

Regardless of sinus symptom improvement, eliminating the underlying dental infection is critical to prevent the possible spread of microorganisms beyond the sinonasal system, both locally and systemically.

## Data Availability

No new data were created or analyzed in this study.

## Abbreviations

The following abbreviations are used in this manuscript:

AI	Artificial intelligence
CT	Computed tomography
ESS	Endoscopic sinus surgery
MA	Maxillary antrostomy
OAC	Oroantral communication
OAF	Oroantral fistula
ODS	Odontogenic sinusitis
SNOT-22	Sino-Nasal Outcome Test-22
